# Modeling and controlling the body in maladaptive ways: an active inference perspective on non-suicidal self-injury behaviors

**DOI:** 10.1093/nc/niad025

**Published:** 2023-11-27

**Authors:** Barca Laura, Domenico Maisto, Giovani Pezzulo

**Affiliations:** Institute of Cognitive Sciences and Technologies, National Research Council, Via San Martino della Battaglia 44, Rome 00185, Italy; Institute of Cognitive Sciences and Technologies, National Research Council, Via San Martino della Battaglia 44, Rome 00185, Italy; Institute of Cognitive Sciences and Technologies, National Research Council, Via San Martino della Battaglia 44, Rome 00185, Italy

**Keywords:** non-suicidal self-injuries, intolerance of uncertainty, interoception, adolescence, active inference

## Abstract

A significant number of persons engage in paradoxical behaviors, such as extreme food restriction (up to starvation) and non-suicidal self-injuries, especially during periods of rapid changes, such as adolescence. Here, we contextualize these and related paradoxical behavior within an active inference view of brain functions, which assumes that the brain forms predictive models of bodily variables, emotional experiences, and the embodied self and continuously strives to reduce the uncertainty of such models. We propose that not only in conditions of excessive or prolonged uncertainty, such as in clinical conditions, but also during pivotal periods of developmental transition, paradoxical behaviors might emerge as maladaptive strategies to reduce uncertainty—by “acting on the body”— soliciting salient perceptual and interoceptive sensations, such as pain or excessive levels of hunger. Although such strategies are maladaptive and run against our basic homeostatic imperatives, they might be functional not only to provide some short-term reward (e.g. relief from emotional distress)—as previously proposed—but also to reduce uncertainty and possibly to restore a coherent model of one’s bodily experience and the self, affording greater confidence in who we are and what course of actions we should pursue.

Highlights

- Predictive processing theories assume that the brain forms internal body and self-models at multiple levels of detail and strives to reduce their uncertainty.
- High levels of uncertainty in internal models of the body and the self are common across several clinical conditions.
- Excessive levels of uncertainty might also be experienced in the typical population during development transitions.
- This excessive uncertainty might hinder the maintenance of a coherent model of the  embodied self and confidently engage in adaptive courses of actions.
- Maladaptive behaviors, such as non-suicidal self-injury, might emerge as paradoxical but effective strategies to “act on the body” to reduce uncertainty

## Introduction

Why do some persons engage in paradoxical behaviors, such as extreme food restriction (up to starvation) and non-suicidal self-injuries (NSSIs)? These and other pathological behaviors are transversal to various psychopathological conditions. Self-destructive actions including self-harm have been associated with post-traumatic stress disorder (Moeller et al. 2001, Yehuda 2002, Weiss et al. 2015, Zelkowitz et al. 2023), eating disorders (Sagiv and Gvion 2020), and borderline personality disorder (Reichl and Kaesset, 2021). Whether paradoxical behaviors as NSSI have a common origin across such and other conditions remains to be systematically tested, which is particularly challenging as—in most cases—they can only be assessed using retrospective measures, rather than measured during their occurrence or induced experimentally. Although they are typically associated with some form of psychopathology, they are not exclusive to clinical conditions but might also be transiently acted by non-clinical individuals. For example, self-injury behaviors are not uncommon during adolescence and progressively decrease in adulthood (Laugesen et al. 2003, Nock et al. 2009, Thielsch et al. 2015, Osmanağaoğlu et al. 2018).

Scholars from a variety of disciplines have questioned why people intentionally harm themselves (either by starving or cutting), proposing various theoretical models empirically tested over time (Chapman et al. 2006, Nock and Mendes 2008a, Nock et al. 2009, Wolff et al. 2019), but we still lack a mechanistic understanding of such behaviors. Here, we propose that despite starvation and NSSI being undoubtedly distinct behaviors with different affective, cognitive, and neurobiological underpinnings, they could share a common “rationale.” Namely, they could be attempts to modify one’s state—in a broad sense, which includes not just the bodily state but also affective, cognitive, and social experiences—by “acting on the body.” In other words, these behaviors can be considered similar because they explicitly target the body, with self-induced hunger or painful sensations, to modify bodily and interoceptive experiences. This perspective helps contextualize harmful behaviors within an “interoceptive inference” framework, which assumes that (interoceptive) bodily sensations and their regulation are key for affectivity, mental health, conscious processes, and the self (Craig 2002, Seth et al. 2012, Barrett and Simmons 2015, Pezzulo et al. 2015, Paulus et al. 2019, Quigley et al. 2021), which could provide novel insights on why people do things to harm themselves intentionally.

A central theme of inferential models of cognition, like active inference, is that actions are motivated by both “utilitarian” imperatives, such as reward achievement, and the “epistemic” imperative to reduce uncertainty about one’s state (intended in a broad sense, from bodily state to one’s identity and self-models). Various researchers proposed that paradoxical behaviors could be motivated by reward achievement since they have (paradoxically) positive effects, such as relief from emotional distress and negative affect (Nock and Prinstein 2004, Chapman et al. 2006, Bresin and Gordon 2013, Selby et al. 2019). Here, we advance a complementary perspective by suggesting that these behaviors might appear less paradoxical when considering that they could serve uncertainty minimization imperatives. For example, we recently suggested that the starvation observed in restrictive anorexia nervosa could serve the imperative of minimizing “interoceptive uncertainty” or the uncertainty about one’s interoceptive state—stretched up to the uncertainty of the self—which might be particularly severe when people receive ambiguous interoceptive signals from the body, when they have poor models of their bodily or emotional state, or when they are particularly intolerant to high levels of uncertainty (Barca and Pezzulo 2020).

What are the possible causes of interoceptive uncertainty? Consider, e.g. a person with physiological deficits in interoceptive pathways that do not allow her to clearly sense interoceptive streams and identify her emotional states and embodied self. These deficits might prevent the person from developing an appropriate model of her emotions (e.g. a model that clearly specifies what emotion she feels and in which conditions) and a good understanding of the emotions and the affective states of others and hence be constantly uncertain about them (see also Smith and Lane 2015, Smith et al. 2018, 2019). Such detachment from one’s body and bodily information might result from adverse experiences during infancy and childhood, such as emotional and physical neglect (Schmitz et al. 2023), or—in less dramatic situations—from reduced affective reciprocity during parental interactions (Conradt and Ablow 2010). Development theories underscore the role of parental care in shaping the experience of self and others and integrative processes of consciousness (Bowlby 1997, Liotti 2004, 2006, Fonagy et al. 2023).

During infancy, a child starts making sense of her internal experiences through the information she gets from the external world, most notably from caregivers whose behavior has a fundamental regulatory function shaping emotional development, stress physiology, and refinement of limbic circuitry (Gee 2016). In addition to the quality of caregivers’ response to the infant’s need for proximity, its “predictability” supports the development of emotions’ regulatory capacity (Gee and Cohodes 2021; Wu and Feng 2020) and a cohesive sense of self (Arciero and Bondolfi 2009), increases prosociality (Deneault et al. 2023), and influences the development of social brain structure (see Ilyka et al. 2021 for a review). Self-report assessment of exposure to unpredictability during early life appears to predict symptoms of anxiety, depression, and anhedonia in adulthood (Glynn et al. 2019). Evidence from cross-species studies indicates that the predictability of caregivers’ behavior in rodents may specifically influence the offspring’s development of corticolimbic circuitry involved in emotion-related functioning (Glynn and Baram 2019). Rodents exposed to unpredictable maternal care exhibit atypical amygdala functioning (Malter Cohen et al. 2013) and weaker connectivity with the medial prefrontal cortex (Guadagno et al. 2018).

Abraham et al. (2019) evaluated a number of features of the neurobiological interoceptive circuit (e.g. the functionality of the amygdala, insula, and oxytocinergic system) in parents and children over the first 6 years of parenthood. Results revealed a critical association between parental interoceptive sensitivity—indexed, e.g. by increased bilateral activation of the anterior insula in response to a video of his/her interacting with his/her infant—the consolidation of the child’s interoceptive circuit and mental health. Taken together, thus, consistent evidence indicates that parental ability to respond appropriately to the children’s needs and bodily signals supports the child’s ability to adequately represent his/her internal bodily states, concurring in the development of self-processes (Fotopoulou and Tsakiris 2017, Ciaunica et al. 2021a, 2021b). The degree of predictability of caregivers’ response appears to be critical for the development of affect regulation and a cohesive sense of the self (Ilyka et al. 2021). When caregivers’ behavior is less reliable, children have more difficulties in distinguishing their own internal states, making self-other distinctions (Ogawa et al. 1997, Dutra et al. 2009), and—in the most severe cases—developing an integrated sense of the self (Liotti 2004, 2006).

As a consequence of these or other deficits in developing appropriate models of emotional and self-models, a person might experience significant interoceptive uncertainty and perceive her own internal states in confused and uncomfortable ways later in life. Suppose that the interoceptive channels are unreliable and the internal models rooted in bodily experiences are poor. In that case, a person might construe a sense of personal stability through external, non-interoceptive signals, such as feedback from others and from the world, rather than via interoceptive signals. Engaging in social interactions, in which we experience affective states relevant to our self-confirmation (e.g. a sense of acceptance and kindness), might be particularly challenging for this person (Guidano 1987, Arciero and Bondolfi 2009). While interacting with others, she might experience ambiguous bodily and emotional states. She might be unable to reduce this uncertainty using the other as an external point of reference since the affectivity attributed to the others might also be perceived as vague and misinterpreted (e.g. “Is he/she interested in me or not?” “Am I a person worthy of attention and love from others?”). Bodily illusions, such as the “rubber hand illusion” (Suzuki et al. 2013, Crucianelli et al. 2018) and the “enfacement illusion” (Sforza et al. 2010, Tajadura-Jiménez et al. 2012), provide compelling evidence for the malleability of self-other boundaries as a function of bodily information and of interoceptive sensibility.

Dealing with these ambiguous situations could be particularly challenging and distressing for a person with no adaptive strategies to reduce uncertainty. Therefore, in these (admittedly extreme) conditions, even maladaptive strategies such as starvation that reduce uncertainty and render bodily and interoceptive stimuli more salient might become more appealing (see also Linson et al. 2020). In other words, while starvation would still be considered paradoxical—in the sense that it runs against the “utilitarian” imperative of ensuring well-being and survival—it might play a functional role for an organism that simultaneously tries to “maximize utility and minimize uncertainty,” as assumed by active inference.

In this article, we propose that NSSI and other kinds of paradoxical behaviors might also be conceptualized in similar ways, i.e. as other cases in which a person intentionally changes her bodily and interoceptive sensations, in order to reduce excessive bodily and interoceptive uncertainty (but also more broadly to modulate affective and physiological states that are otherwise dysregulated, e.g. to decrease the excessive intensity of bodily sensations in hyperarousal).

In the next sections, we first discuss NSSI behaviors by focusing on the fact that they might occur in non-clinical individuals. We will highlight that this could be not only especially the case during adolescence—a period of life during which people experience various kinds of uncertainties (e.g. their bodies, the self, their social status, and interpersonal relationships), but also more speculatively during other periods of life associated with substantial changes and uncertainty, such as the perimenopause–menopause transition period in women. Then, in the subsequent section, we introduce the main tenets of active inference by focusing on its proposed mechanisms for interoceptive processing and uncertainty reduction. Finally, we discuss how the active inference framework might help conceptualize NSSI as a possible strategy to reduce uncertainty; for example, the uncertainty that some (non-clinical populations of) adolescents might strive to cope with during their transition to adulthood.

## Paradoxical behaviors: the case of NSSI

“Self-injury behaviors” is an umbrella term that includes a wide range of behaviors (and intentions), including suicide attempts, superficial cuts, and medication withdrawals (Skegg 2005, Nock 2010). We focus on NSSI behaviors as the direct, deliberate destruction of body tissue without lethal intent (e.g. cutting oneself). A distinction is also made between NSSI performed stereotypically in the context of developmental disabilities (e.g. head banging) and major injuries often observed in psychotic disorders. The most frequent examples of NSSI include cutting the skin with a sharp object (e.g. a knife, razor blade, or needle) and skin burn, usually with a cigarette (Khanipour et al. 2016). Patients often injure themselves, in a single act, by inflicting multiple injuries at the same body site, usually in areas that are easily hidden but accessible (e.g. forearms and anterior thighs). The behavior is often repeated, resulting in extensive scarring patterns. The age of onset of NSSI tends to be early adolescence, between 12 and 14 years of age, and the behavior appears to decline after young adulthood (Nock et al. 2009).

The phenomenology of NSSI is still elusive. It has been hypothesized that self-injury behaviors might be engaged to regulate emotions by avoiding or distracting from unwanted emotional experiences (e.g. shame) or negative beliefs about the self (Chapman et al. 2006). This “affect regulation hypothesis” is supported by the findings that emotional dysregulation—such as reduced emotional clarity, problems in goal-directed behaviors, and impulse control—is common in non-clinical samples of adolescents who engage in NSSI. The probability of engaging in self-injuries appears to be positively associated with emotional dysregulation, particularly when individuals lack more adaptive strategies (e.g. reappraisal) to cope with their affectivity (Wolff et al. 2019). Likewise, self-injurers typically describe their behaviors as attempts to avoid negative cognitive (i.e. bad thoughts or memories) and affective (i.e. anxiety, sadness, and anger) states, whose elevated arousal is suggested to increase the likelihood of engaging in self-injuries (Nock and Mendes 2008b, Nock et al. 2009). The interplay between physiological hyperarousal, poor distress tolerance, and reduced problem-solving skills is thought to concur in self-injury behaviors (Nock and Prinstein 2004, Nock et al. 2006), with adolescents with a history of NSSI displaying greater changes in skin conductance and reduced tolerance to increasingly stressing conditions (Nock and Mendes 2008b).

A factor that might contribute to the genesis of maladaptive behaviors—including NSSI—is the extent to which one is able to sustain the rise in the levels of uncertainty, which, broadly speaking, might occur in different domains of subjective experience (from bodily states to the possible outcomes of social interactions, to the self, and to one’s personal identity). Every day, we face some kind of uncertainty, but the degree to which it might be distressing largely varies across individuals. High levels of intolerance of uncertainty, or the attitude to react negatively to uncertain situations and events (Ladouceur et al. 2000), are considered a transdiagnostic vulnerability factor across several clinical conditions such as anxiety, depression (McEvoy and Mahoney 2012), and eating disorders (Kesby et al. 2017). Recently, such “fear for uncertainty” has also been reported in neurotypical young adults and adolescents in relation to excessive worry (Laugesen et al. 2003, Thielsch et al. 2015) and anxiety (Osmanağaoğlu et al. 2018). There is also evidence of a positive reciprocal association between intolerance of uncertainty and difficulties in emotion processing in adolescents and that both tend to decrease with the transition to adulthood (Lauriola et al. 2023). Crucially, NSSI is particularly prominent in adolescence; its rate in non-clinical samples of adolescence has increased alarmingly in this century, representing a serious ongoing societal health concern (Xiao et al. 2022).

## NSSI in adolescence

Adolescence is the period of developmental transition from childhood to adulthood, which might be stretched up to the early 20s due to current sociocultural changes (e.g. delays in completing education, occupational attainment, and parenthood) (Patton et al. 2018). Among the challenges that adolescents have to face are the structuring of a “narrative identity” or self-story, featuring the development of a sense of personal identity that integrates past experiences with current, and future goals and meanings in a coherent whole over time (McAdams and McLean 2013, McLean and Lilgendahl 2019). The definition of the new boundaries of adolescents’ personal identity involves significant changes in the reciprocity with caregivers and peers. Thus, in parallel to the negotiation of identity with caregivers (through a relative detachment from them, a renegotiation of intimacy, and the questioning of their confirmatory authority), the modifications of friendship structures—from childhood to adolescence—lay the ground for the progressive recognition of social contexts and peer relationships as the elite territories for the modulation and exploration of personal identity. The redefinition that the adolescent has to face in these territories of exploration (of the self as an individual separated from the other and of the self with the other) might pass through a phase of reduced coherence in the narration of the self and hence an increased level of uncertainty. Coherence in the self’s narrative is considered a measure of well-being and has been associated with psychopathology in adulthood (Klimstra and Denissen 2017) and adolescence (Lind et al. 2020, Shiner et al. 2021). For example, narrative incoherence has been found to be associated with personality disorders in adolescents (Lind et al. 2019), where “identity diffusion” (e.g. feelings of emptiness and being fragmented and lack of a sense of continuity over time) might be considered an expression of high levels of uncertainty of the self.

Emotion-wise, a developmental trend toward an increased specificity of emotion-related maps of bodily sensations (Barca et al. 2023)—a proxy of interoceptive representations of emotions—has been reported from children aged 6 years to adulthood (Hietanen et al. 2016). Pubertal changes encompass dramatic bodily and neuroendocrine system changes, comprising—but not reduced to—changes in the reproductive, adrenal, and growth axes (Cameron 2004). Thus, adolescents might face at least four sources of uncertainty: (i) the uncertainty due to physiological alterations related to bodily changes and to modification in hormonal levels leading to sexual maturity; (ii) the uncertainty in self-identity (i.e. the structure of self-awareness) and personal identity (i.e, the narrative diachronic self) (Drummond 2021), which might be coupled with changes in body image and the development of gender identity; (iii) the uncertainty in affect regulation, with the emergence of new forms of affectivity as feelings of love and sexual attraction toward a partner; and (iv) uncertainty in the social context, with respect to their social status and role expectations in the adult society. Such high levels of uncertainty might lead to a poorly defined sense of self, with unclear boundaries and a sense of emptiness. In this context, pain becomes a possible way to recover a bodily sense of self, and self-injurious behavior might be instantiated as an attempt to reduce the rise in the levels of uncertainty in these (and potentially other) domains, toward the transition to adulthood (see Miller et al. 2020 for a closely related approach on addiction).

## Active inference, interoceptive processing, and uncertainty reduction

Active inference is based on the idea that in order to engage in adaptive allostatic regulation and goal-directed behavior, living organisms continuously strive to minimize the surprise of their sensations or, more formally, an upper bound to surprise: variational free energy (Parr et al. 2022). Notably, the (expected) free energy minimization processes that drive active inference jointly consider two complementary objectives. The former (utilitarian) objective is to realize one’s preferences, such as being satiated or safe, by minimizing the discrepancy between preferred sensations (encoded as “priors over observations” in active inference) and current sensations in different modalities (e.g. interoceptive or exteroceptive). The latter (epistemic) objective is to reduce uncertainty about one’s estimated state. This means that active inference agents tend to avoid ambiguous states, encompassing the avoidance of ambiguous places where self-localization is challenging, ambiguous social situations where safety is uncertain, and ambiguous bodily states, such as unsure feelings of fatigue. However, one apparent exception to this aversion to ambiguity arises when exploring novel states implies the opportunity to learn new things and enhance one's model; see Friston et al. (2017) for a discussion. Furthermore, and importantly, active inference agents will actively operate in the environment to reduce their ambiguity; for example, by actively seeking informative sensations that disambiguate in which location they are (e.g. by looking for traffic signs), whether their social context is safe or unsafe (e.g. by trying to understand other’s intentions from their facial expressions and actions), or whether they are currently fatigued (e.g. by putting attention to one’s heart), happy, or sad.

The last examples—disambiguating one’s fatigue and emotional states—may seem strange if one assumes that we do have direct access to the body- and allostasis-related states (e.g. states of satiation, thirst, and fatigue) and to our emotions (e.g. we automatically know whether we are happy or sad). However, one assumption of active inference is that one’s bodily and emotional states are not necessarily observable but, instead, “hidden states” that need to be inferred on the basis of sensations (especially, but not exclusively, of interoceptive sensations from the inside of the body) and of an implicit, unconscious model of how the body functions (Barrett and Simmons 2015, Pezzulo et al. 2015, Seth and Friston 2016). In other words, the same inferential process that allows active inference agents to estimate the hidden state of the external environment (e.g. the presence or absence of an object in the environment) is also used to estimate other hidden states, such as fatigue, happiness, or sadness. This implies that one can also be wrong, or be fooled, about these states; for example, we could experience the “interoceptive illusion” of feeling more fatigued than our physiological parameters would afford (Iodice et al. 2019).

**Figure 1.**
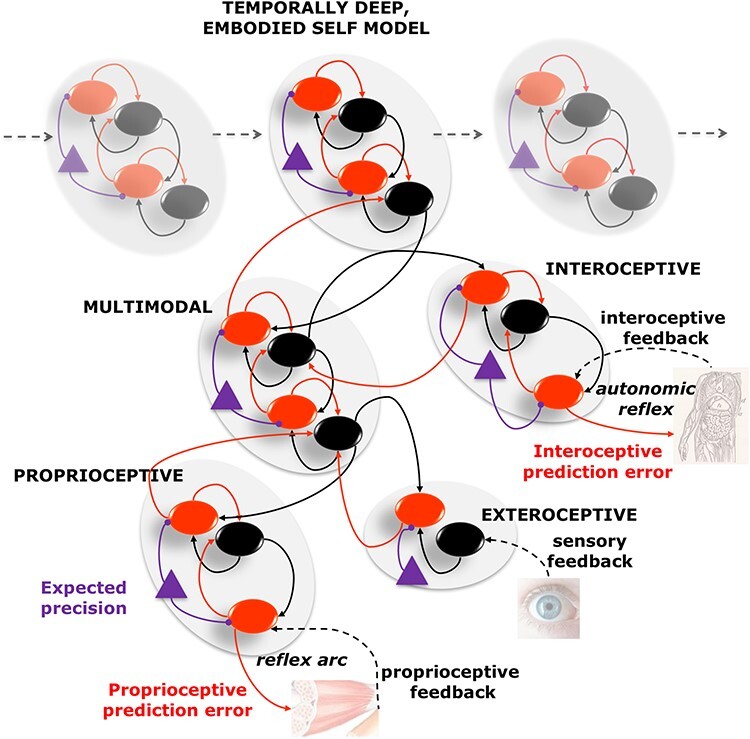
A schematic illustration of a hierarchical active inference model. This model links (exteroceptive, interoceptive, and proprioceptive) sensations at lower levels with multimodal models of hidden bodily states, such as fatigue and hunger, at intermediate levels, and finally with temporally extended, integrative models of the embodied self at the higher hierarchical level. In this schematic, following predictive coding (Rao and Ballard 1999, Friston 2005), black and red circles represent neural units that encode predictions and prediction errors, respectively. The levels are reciprocally connected, so predictions are propagated from the top-down (black edges) and prediction errors from the bottom-up (red edges). Finally, the pink triangles indicate a mechanism of precision gating (or gain control) of prediction error units, which determines their relative influence on units encoding predictions. At a neurobiological level, prediction and prediction error units could be mapped to deep and superficial pyramidal cells in cortical hierarchies, whereas expected precision could be linked to neuromodulatory input. The elements of the generative model shown do not need to map one-to-one to specific brain areas or networks but are plausibly distributed across many of them. However, as a first approximation, the lower and intermediate layers of the generative model could be linked to brain networks that process unimodal information (e.g. sensory cortices for exteroceptive information) and multimodal association areas, respectively. The highest level of the generative model could be linked to brain networks that process information about the self, such as the insular cortex, the anterior cingulate cortex, and the medial prefrontal cortex. See Parr et al. (2022) for details about hierarchical generative models supporting adaptive regulation and allostasis and Barrett and Simmons (2015) for their putative neuronal underpinnings. See online article for colored version of this figure.

Extending this idea even further, one can assume that certain emotional states, as well as self-awareness and the (embodied) sense of self—and the feeling of continually being the same person—could be constructed similarly: it would be the result of an inferential process that integrates bodily sensations and other experiences over time (Gu et al. 2013, Seth 2013, Stephan et al. 2016, Barrett 2017). Figure 1 illustrates graphically this perspective by showing a (schematic) hierarchical generative model that links (exteroceptive, interoceptive, and proprioceptive) sensations at lower levels with multimodal models of hidden bodily states, such as fatigue and hunger at intermediate layers, and, finally, with temporally extended, integrative models of the emotional and embodied self at the higher hierarchical level. The hierarchical generative model recapitulates a simple predictive coding architecture, which includes various putative brain areas or networks (gray ovals) arranged hierarchically. In the schematic, networks for unimodal (exteroceptive, proprioceptive, and interoceptive) processing are situated at the lowest hierarchical level, multimodal networks are at an intermediate level, and networks for processing a persistent model of the self are at the highest level. Note that this simple schematic is not supposed to recapitulate brain anatomy but to illustrate the basic principles of hierarchical generative models and predictive coding; (for a discussion of the mapping between predictive coding networks and brain anatomy, see Parr et al. 2022). Each network includes cells encoding predictions (black nodes) and prediction errors (red nodes). These units are reciprocally linked through top-down connections that convey predictions (black edges) and bottom-up connections that convey prediction errors (red edges), within and across levels. This predictive coding architecture permits inferring (in the Bayesian sense) the most likely causes of sensations, across multiple modalities and multiple hierarchical levels, by minimizing prediction errors at all levels. The rationale is that predictions at all levels are continuously adjusted (and synaptic weights adjusted at a slower time scale) until they match with incoming multimodal stimuli sufficiently well, and, consequently, the prediction errors across all levels are minimized. This process entails that even if a predictive coding agent starts with an incorrect prediction (e.g. about what object it is looking at) the prediction errors that measure a discrepancy between the predicted sensations and the actual sensations can help revise the initial predictions. See Parr et al. (2022) for a more detailed explanation of how to interpret these schematics.

Another critical aspect of Fig. 1 is that it illustrates two pathways in which prediction errors at the proprioceptive and interoceptive levels are used to steer physical actions (reflex arcs) and autonomic actions (autonomic reflexes). Endowing predictive coding with these reflexes—hence realizing an “active inference” architecture—permits minimizing prediction errors by changing the state of the world (by physically acting) or the internal milieu (by engaging in autonomic actions) rather than only by changing predictions, as described later.

Equipped with a generative model like the one shown in Fig. 1, an active inference agent can continuously infer (and act upon) the state of the world and of the body, including the internal milieu, at multiple time scales. Of particular interest, here are multimodal inferences that unite exteroceptive and interoceptive sources of evidence. One example of this is the perception of faces expressing emotions. Two studies reported that participants processed faces expressing fear (but not neutral faces or faces expressing other emotions) when their heart rate was high—hence congruent with the fearful expression (Pezzulo et al. 2018, Yu et al. 2021). The generative model shown in Fig. 1 could support this kind of inference by using interoceptive information from the heart (i.e. high heart rate) as evidence that “there might be something fearful out there” (Pezzulo 2013). Another more complex example regards emotional awareness and self-awareness—which significantly engage the brain regions involved in interoception and the representation of physiological processes (Garfinkel et al. 2013). The generative model shown in Fig. 1 might support processes of emotional awareness in a way that is neither purely bottom-up (i.e. as if interoceptive signals cause emotional awareness) nor top-down (i.e. as if emotional awareness causes interoceptive signals), but rather through a circular causality between central predictions about bodily state—that engage autonomic reflexes—and interoceptive streams—that update the predictions (Seth and Friston 2016). In this perspective, any representation that induces interoceptive predictions could be associated with emotional or affective content; crucially, this is also the case with some aspects of self-awareness (e.g. recognizing one’s own face) that require integrating interoceptive streams with concurrent exteroceptive (e.g. visual) and proprioceptive cues. These examples illustrate that the generative model of Fig. 1 natively implements both the multisensory integration required to unite (for example) interoceptive and exteroceptive streams and the active aspects that are supposed to support emotional and self-processing—and the construction of an “embodied self” (i.e. the circular causality between engaging autonomic reflexes and capturing the ensuing interoceptive signals).

In general, the accuracy of the inference of hidden bodily states, the “embodied self,” or other aspects of the model depends on the signal-to-noise ratio of the sensations and on the quality of the model. For example, it is difficult to self-localize in a city if it is dark (low signal-to-noise ratio) or if one does not know the city well (poor model). The inference of hidden bodily and emotional states might function in an analogous manner. If the quality of the afferent interoceptive (e.g. cardiac) signals is low, or if one has a poor model of how one’s body functions, then it would estimate one’s bodily states such as fatigue incorrectly (which in turn would also impair its adaptive regulation of the same bodily states). Interoceptive signals could be “too noisy” for various reasons, which might be related to physiology, inflammation, or stress. The body model can be poor in various ways, too. For example, it could poorly characterize the statistical relations between interoceptive sensations and hidden bodily states (e.g. systematically mischaracterize high heart rate as caused by hunger but not fatigue or joy).

Finally, there is a third essential element that determines the accuracy of the inference: precision control. In predictive coding, the influence of prediction errors on inference is weighted by their precision, i.e. inverse variance (pink triangles in Fig. 1). This weighting would ensure that very reliable sensations have more impact on inference than unreliable sensations. However, precision (like all other variables) needs to be estimated, but this might be incorrect. An incorrect setting of precisions has been associated with various psychopathological conditions, such as psychosis (Adams et al. 2013), eating disorders (Barca and Pezzulo 2020), panic disorders (Maisto et al. 2021), symptom perception (Pezzulo et al. 2019), depression (Barrett et al. 2016), and many others (Khalsa et al. 2018, Paulus et al. 2019). Intuitively, assigning excessively high weight to noisy sensations yields an incorrect inference that tracks the noise rather than the correct state of the estimated variable system (i.e. overfitting), whereas assigning excessively low weight to sensations (or excessively high weight to prior knowledge) makes the system poorly responsive to incoming observations that might signal a change in the state of the system—and both are examples of aberrant inference (Friston et al. 2014).

**Figure 2.**
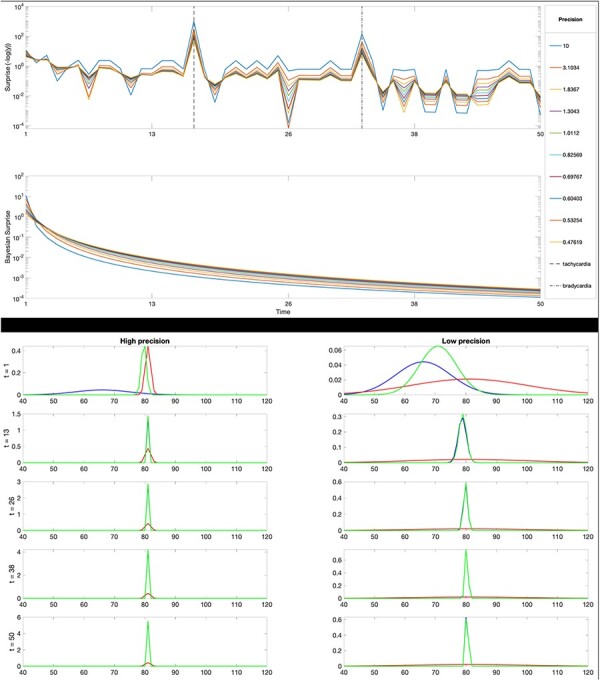
A simplified example of (Bayesian) inference of one’s heart rate. First panel: simulated time series of heartbeat observations. Second panel: Shannon surprise of a generative model composed of a fixed prior about heart rate (a Gaussian with a mean of 67 and a precision of 0.11) and a likelihood (a Gaussian centered on the current heart rate with an additional bias of 15 pulses, with various precisions that vary between 0.47 and 10, see the legend). Third panel: Bayesian surprise, which measures the discrepancy between posterior and prior probabilities over time. Bottom panels: the two series of panels are organized in two (left and right) columns, which show the first five time steps of inference for the two cases with high precision (of 10) and low precision (of 0.1) of the likelihood, respectively. See the main text for an explanation and online article for colored version of this figure.

Figure 2 provides a formal illustration of the above by plotting some examples of Bayesian inference using generative models under various levels of precision of the model components. For simplicity, we focus on a simplified example of inference of an interoceptive variable: one’s heart rate. Heart rate is a “hidden variable” in Bayesian parlance since it is not directly observable but needs to be inferred through two sources of information: prior knowledge about the most likely heart rate and sensory (heartbeat) observations. The top panel of Fig. 2 shows a series of (noisy) heartbeat observations. In the beginning, they are in the normal range for an adult (time steps 1–10), then they increase significantly, simulating tachycardia (time steps 11–20), then they go back to the normal range (time steps 21–30), then they decrease significantly, simulating bradycardia (time steps 31–40), and finally, they go back to the normal range (time steps 41–50).

The second panel of Fig. 2 shows the Shannon surprise of an inference model that estimates the current heart rate using the two standard components of a generative model. The former component is the prior, which encodes the person’s a priori probabilistic belief (i.e. probability distribution) about her “normal” heart rate range; here, the prior is a Gaussian centered on 67 and has a precision of 0.11. The latter component is the likelihood, which encodes the probabilistic mapping between sensory (heartbeat) observations and the hidden state (heart rate); here, the likelihood is a Gaussian centered on the current heart rate with an additional bias of 15 pulses, and the panel shows the results for 10 values for precision obtained by subdividing the range [0.1,10] into equal intervals. The results shown in the second panel of Fig. 2 show that Shannon surprise increases dramatically during episodes of tachycardia and bradycardia, which are far from the normal range. The pattern of results is the same across all levels of likelihood precision. However, the inference with a very high precision (a precision of 10) tracks more closely the noise sensory signals and can therefore lead to more extreme results.

The third panel shows the Bayesian surprise (or the Kullback-Leibler divergence between posterior and prior probability distributions) over time. This is a measure of how much dissimilar the posterior and the prior are, and it always decreases as a result of inference, but note that it decreases much more rapidly when the precision of the likelihood is 10, which is another indication that the posterior is “overfitting,” meaning that the inference result is excessively biased by the likelihood distribution.

Finally, the two bottom series of panels are organized in two (left and right) columns, which show the first five time steps of inference for the two cases with high precision (of 10) and low precision (of 0.1) of the likelihood, respectively. In these plots, the prior distributions are in blue, the posterior distributions are in green, and the likelihoods are in red. It is possible to note that in the left (high precision) panels, the posterior inference closely follows the likelihood (it “overfits”) after five time steps and the inferred heart rate is slightly biased (i.e. it is 79). Differently, in the right (low precision) panels, the inference converges much slower to a high precision posterior, but without overfitting.

These simple examples of Bayesian inference illustrate two things. First, sensory observations that are unpredictable given the current model generate significant surprise, and sometimes, the surprise can remain relatively high for long periods before the model adapts (or the world changes), especially with some parameterizations of the generative model. This is particularly relevant in this context since active inference agents strive to minimize their surprise (and the long-term average of surprise, entropy, which is a measure of uncertainty) by changing their model, or changing the world, or both.

Second, these examples illustrate the importance of precision control and the appropriate setting of precision parameters in guiding inference. Remarkably, the inference can be more or less accurate or fast using the same data, depending on the precision parameters. Note that in Fig. 2, we manipulated only the precision of the likelihood. However, it would also be possible to manipulate the precision of the prior, together or in alternative to the precision of the likelihood. Generally speaking, when the precision of the prior is very high, the posterior will closely reflect the prior, rendering the inference rigid and incapable of adapting to changing environmental conditions—which might be especially problematic in periods of significant changes, such as adolescence or more simply when one changes city, working environment, and friends. Furthermore, as shown in Fig. 1, hierarchical predictive coding architectures have precision values associated with every hierarchical level (whereas, for simplicity, the inference shown in Fig. 2 is not hierarchical). The correct balance of precision parameters within and across layers is crucial for accurate inference, as it ensures that the correct levels of confidence are assigned to data and prior information.

Finally, and importantly, aberrant precision control (as well as various combinations of other factors discussed earlier, such as noisy bodily sensations and poor bodily mode) can render inference not just incorrect but also highly ambiguous, leaving a person in a permanent condition of uncertainty about whether one is fatigued (when considering the bodily state), happy, or sad (when considering the emotional state), what kind of person one is or what are one’s desires (when considering self-models), etc. Importantly, this condition of uncertainty is not limited to perceptual inference but has a cascade effect on decision-making and action selection. Indeed, an uncertain estimate of one’s state automatically implies that one has low confidence in the effects of one’s plans; for example, it renders more difficult the prediction of whether a run would be too fatiguing or a party too stressful. It is exactly this kind of uncertainty (about the present and the future, the body state or the outcomes of social interactions, etc.) that active inference agents strive to avoid.

### Avoiding excessive uncertainty in maladaptive ways

Our previous discussion clarified that active inference agents have sophisticated (hierarchically deep, temporally extended) models of themselves that permit making inferences at multiple levels about hidden bodily states (which comprise both the classical “body schema” and other states that are relevant for allostasis, such as hunger, thirst, and fatigue) and other states related to the emotional and embodied self. These models are essential for ensuring effective regulation and control at multiple levels, from simple reflexes to sophisticated goal-directed behaviors (Tschantz et al. 2022). However, in some cases, the aforementioned inferential process might not work properly (e.g. if the sensory channels are too noisy or are assigned excessively high or low precision). As a consequence, a person could experience an excessive or irreducible uncertainty about her bodily and emotional states or about the self, which in turn translates into a loss of confidence about which future courses of action could produce desired outcomes. Crucially, active inference agents follow the imperative to avoid such an uncertainty about the present or the future. Normally, uncertainty minimization strategies are adaptive (e.g. seeking advice if one is uncertain about the direction of the preferred restaurant). However, in some conditions, such as when a person experiences excessive and irreducible uncertainty and when the uncertainty is particularly distressing or related to fundamental life concerns, she might potentially seek “maladaptive” ways to reduce it—or methods that reduce uncertainty at the cost of hindering fundamental imperatives of well-being and survival (see also Linson et al. 2020).

In this perspective, apparently paradoxical actions, such as food restriction and self-injurious behaviors, might be pursued because they could contribute to reducing the (otherwise unmanageable) uncertainty about bodily and emotional states or the self. In other words, in some conditions, the self-injuring pain could be more than compensated by the information gain—and the possibility to generate precise sensations about one’s bodily state. By harming the body, we turn it into a very precise source of sensations that relieves us from excessive uncertainty about the present state and the future course of action. Our (simple) example, therefore, illustrates a possible way paradoxical actions could be pursued by active inference agents who endure to minimize their uncertainty. While self-injuries and other similar behaviors are maladaptive in the sense of reducing the fitness of an organism, they can still emerge as a result of a correct inference that tries to minimize the uncertainty of one’s model of the body and the self. This case could particularly fit when some of the (precision) parameters of one’s model of the body and the self are not appropriately tuned (Fig. 2), producing excessive levels of uncertainty.

Having said this, the idea that NSSI behaviors could reflect the imperative to minimize uncertainty is not at odds but complementary to the idea that these behaviors might also be motivated by reward achievement (remember that in active inference, both uncertainty minimization and utility maximization can be in play simultaneously). While NSSI behaviors are associated with a variety of adverse outcomes, such as negative emotions and distress (Klonsky et al. 2003), they can also have paradoxically positive effects by providing a way to relieve or distract from other sources of emotional distress and negative affect (Nock and Prinstein 2004, Chapman et al. 2006, Bresin and Gordon 2013, Selby et al. 2019). The hedonic effect of NSSI behaviors might be further magnified by poor models of one’s body and the self, as suggested by evidence that children who engage in NSSI show aberrant responsiveness to rewards (Tsypes et al. 2018). Finally, NSSI behaviors have habitual components, which might contribute to their selection, over and above consideration of utility maximization or uncertainty reduction (Magerl et al. 2012). This body of evidence suggests that if uncertainty minimization is a driver of NSSI behaviors, as suggested here, it could work in concert with other drivers (reward achievement and habit), in ways that are still poorly understood.

Focusing on uncertainty minimization as a possible factor contributing to NSSI behaviors might also help understand the prevalence of NSSI during adolescence. As discussed earlier, people in adolescence experience significant changes at many levels—from bodily states such as body size to interoceptive and hormonal processes to affective states and the self. As illustrated in Fig. 2, rapid changes (as in the cases of simulated tachycardia and bradycardia) determine high levels of surprise and uncertainty that, in some cases, remain elevated, either because some of the precision parameters that afford model updates are set incorrectly or simply because readapting internal models of the body and the self takes time. In periods of rapid changes, such as adolescence or after very surprising events, there might be a (temporary) misalignment between the predictions of the (outdated) internal model and the incoming sensations. For example, during adolescence, one might use an outdated model that predicts the usual affective states during a party and fail to contextualize novel sensations (e.g. unexpected feelings or interoceptive signals when meeting somebody), hence experiencing high levels of uncertainty. Thus, failing to reduce this uncertainty and achieve a coherent model of oneself could be particularly distressing.

## Discussion

Current theories of predictive processing and active inference assume that, to steer adaptive perception and action, the brain forms internal generative models of the environment and of the body within it. Various studies reveal that the brain has rich models of the body; for example, it integrates somatosensory and proprioceptive information into a coherent representation of things like body size and limb position—i.e. a “body schema.” More recently, this model-based perspective has been extended to interoception—and the rich sensations we constantly receive from the internal body. Theories of interoceptive processing propose that the brain continuously estimates key bodily and homeostatic variables, such as thirst or fatigue levels, perhaps forming something like an “interoceptive schema.”

A key reason for forming bodily or interoceptive models is that they permit us to exert accurate control over the variety of signals (e.g. somatosensory and interoceptive) that the body produces. Forming an accurate body schema is prominent for motor control, whereas modeling interoceptive variables (e.g. thirst) is key to keeping them under control by engaging autonomic reflexes (e.g. vasodilation) and allostatic or goal-directed actions (e.g. drinking) when they have incorrect values. The generative modeling perspective can also be extended hierarchically to consider richer models of multimodal experiences and “embodied self” that persists in time and anchors our experiences, permitting us to select adaptive courses of action to achieve our favorite goals.

While it seems obvious that controlling bodily variables and achieving goals are crucial for survival, this perspective poses a fundamental challenge. In control theory and active inference, “controlling” the body ensures that the body generates the preferred outcomes with high (hedonic or pragmatic) value, e.g. safe levels for thirst and fatigue. This idea applies naturally to many of our activities that pursue some form of biologically adaptive function or well-being, such as ensuring that we keep our bodies healthy and consume good food (Sterling and Eyer 1988, Sterling 2012). However, it fails to explain why we engage in some activities that are apparently maladaptive and contradict our primary biological imperative to ensure body health. Perhaps the most puzzling examples are pathological behaviors (e.g. non-suicidal self-harm or starvation), which are common across psychopathological conditions. In these cases, the control exerted over the body and its sensations might serve the purpose of generating outcomes with high (hedonic or pragmatic) values that nevertheless run against our homeostatic and survival imperatives (e.g. pain and excessive levels of hunger).

In this article, we started with formal accounts of brain processing based on active inference to discuss the mechanisms and functional purpose of the (apparently) maladaptive ways to “control the body” that arise in these and other psychopathological behaviors. We first discussed how we build models of the world, of our bodily and interoceptive processes, of our emotions, and of the embodied self, which provides a sense of understanding of reality and affords adaptive control at many levels, from the allostatic regulation of our physiological states to the achievement of our individual and social goals. Then, we discussed under which conditions we can become highly uncertain about our current state and the future course of action. These conditions include both contextual factors (e.g. periods of noteworthy changes or stress) and factors related to the person’s internal models (e.g. poor models in which precision parameters are incorrectly set). We next turned to active inference and discussed how reducing uncertainty (not just maximizing utility) is a key imperative in this framework. This implies that an active inference agent can sometimes privilege uncertainty minimization over utility maximization. In extreme conditions, such as when interoceptive uncertainty is excessive or difficult to reduce, a person could develop maladaptive strategies to deal with it, such as acting on the body to produce interoceptive sensations of pain or starvation that reduce interoceptive uncertainty.

The centrality of physiological processes and bodily information for the sense of self has been widely discussed by interoceptive research (Seth et al. 2012, Quigley et al. 2021). Here, in continuity with previous works (Barca and Pezzulo 2020), we suggest that (i) some pathological behaviors—that “act on the body” in maladaptive ways—might be considered as strategies for modifying internal models and the sense of self when it is deficient, through bodily sensations and (ii) the sense of self can be deficient when bodily information is uncertain, and this can happen not only in clinical conditions but also during pivotal periods of developmental transition, e.g. in adolescence.

The theoretical perspective offered here leaves several important questions unaddressed. First, even if uncertainty reduction might be a central drive in self-injury behaviors, it is unclear what kinds of uncertainty (if any) specifically trigger the paradoxical behaviors. It may be only the uncertainty at deep hierarchical levels (e.g. at the level of self-models) that promotes paradoxical behaviors. Alternatively, it could be possible that it is not so much the kind of uncertainty that matters but somewhat its associated distress, which in turn could be amplified by conditions like the intolerance of uncertainty. While these and alternative hypotheses remain to be tested in future research, they might in the future lead to novel tailored interventions. Current reviews of NSSI interventions (see, e.g. Turner et al. 2014, Witt et al. 2021) outline the various treatments currently available (e.g. psychological and psychosocial interventions, pharmacological treatments, and a combination of both), but underline the need for further data on their effectiveness. The use of formal models of brain function to characterize the mechanisms of psychopathology (Friston et al. 2014, Stephan and Mathys 2014) might help conceptualize dysfunctional behaviors in operationalizable terms. In this vein, one might delineate interventions aimed at reducing the uncertainty of self-models by starting from the bodily self and the definition of self-other boundaries (if these turn out to be the critical aspects for the patient). In this endeavor, techniques such as virtual reality and robotics might help elucidate which levels of the multisensory integration process of the bodily self might be compromised (Dieguez and Lopez 2017, Tsakiris 2017, Serino et al. 2018). Virtual reality along with role-playing sessions and the use of avatars are increasingly considered effective tools for the training of clinicians who deal with individuals engaging in NSSI (Taliaferro et al. 2023). It remains to be tested whether the use of virtual reality or similar interventions—and the definition of contexts and tasks aimed at reducing the uncertainty of the bodily self—might also be viable for individuals engaging in NSSI.

Second, in this paper, we have mainly focused on uncertainty reduction, but as we reviewed earlier, there are other alternative (or complementary) perspectives on the genesis of NSSI that considers elements such as affective regulation. In addition to the studies discussed earlier, other insights into the pathological mechanisms that might underlie NSSI come from the analysis of clinical populations. For example, dysregulations of the “endogenous opioid system”—involved in reward and the regulation of pain and affect—have been documented (Bresin and Gordon 2013), as low basal plasma levels of β-endorphins (which are peripherally released following tissue damage) in psychiatric patients (van der Venne et al. 2021, Cakin Memik et al. 2023). Lower salivary levels of β-endorphins have also been registered immediately before NSSI, compared to post-NSSI (Störkel et al. 2021). Thus, an imbalance in the opioid system might be a relevant component in NSSI, where self-injures might be acted to initiate the release of β-endorphins to restore homeostasis (Stanley et al. 2010, Bresin and Gordon 2013). Another line of research examined “nociceptive dysregulation,” with possible hypoalgesia (Kirtley et al. 2016), showing higher pain thresholds and lower pain intensity in adolescents with NSSI (Nock et al. 2006, 2009, van der Venne et al. 2021). Recent works failed to replicate these findings but reported specific alterations in descending inhibitory pain control (Leone et al. 2021, Lalouni et al. 2022). Despite some inconsistencies, this is an area that is worth further investigation. It remains to investigate to what extent the perspective on NSSI offered here could be extended to cover the aforementioned body of evidence and whether active inference could help integrate the different perspectives we have discussed.

We focused on adolescence as a potentially critical period for NSSI, given that it is associated with high levels of uncertainty about several central domains in human life. However, there are other (gender-related) developmental periods in which bodily changes might be coupled with increased levels of uncertainty (e.g. in physiology, in the sense of self, in the social role) and vulnerability. Pregnancy and transition to menopause, e.g. are periods of endocrine and hormonal upheavals that might impact a woman’s affective life and well-being. These physiological changes are coupled with a fundamental developmental transition that requires a redefinition of personal identity and narrative integration (McLean and Lilgendahl 2019), with increased uncertainty of one’s internal states and role in the social context. Taking into account the perimenopausal and menopausal transition, the physiological, psychological, and affective experiences associated with it are very heterogeneous. Some women might experience it as a new beginning, whereas for others, it may be more critical (Deeks 2003). In some cases, e.g. the menopause transition might perturb the continuity of one’s sense of self, inducing discrepancies in internal self-coherence (e.g. the end of childbearing years, the aging process), which might increase the level of distress (Barca and De Marchis 2018).

The dramatic changes that a women’s physiology undergoes during life have been suggested to concur with the atypical interoception often reported (e.g. heightened interoceptive attention but poor interoceptive accuracy), which might contribute to their greater vulnerability to mental illness (Murphy et al. 2019). Although this is still a speculative hypothesis that needs to be tested empirically, the effect of these transition periods on women’s well-being is currently overlooked and deserves more attention.

Finally, although we only focused on “maladaptive” strategies to modify the sense of self through bodily sensations, there are also “adaptive” strategies that use the body to improve the sense of self and feelings of well-being. Among these, e.g. is engaging in physical activities to reduce emotional distress. Performing physical activity concurs in the reduction in symptoms of depression and anxiety, acting on both psychological (e.g. diverting from unpleasant stimuli or increasing the sense of self-efficacy) and physiological mechanisms (e.g. through the release of monoamines and endorphins), which seem to alleviate distressing emotions (see for a review Paluska and Schwenk 2000). The relationship between well-being, physical activity, and interoception is increasingly receiving attention (Wallman-Jones et al. 2021) and deserves further investigation for its potential role as a protective factor against emotional distress and the development of clinical conditions.

It is worth reminding that the theoretical proposal advanced in this study—that NSSI might emerge when some of the (precision) parameters of one’s model of the body and the self are not appropriately tuned—is still speculative. However, previous studies reported the importance of aberrant precision tuning in interoceptive streams across various psychopathological conditions, such as depression, anxiety, eating, and substance use disorders (Smith et al. 2020, 2021). These studies, along with other proposals (Khalsa et al. 2018), raise the possibility that interoceptive dysfunctions and the incorrect tuning of (precision) parameters of generative models might have a pervasive effect on psychopathology. This hypothesis remains to be investigated in the case of NSSI.

## Data Availability

No new data were generated or analyzed in support of this research.
